# Improving the Accessibility and Responsiveness of Domestic Abuse Services

**DOI:** 10.1177/10778012251384632

**Published:** 2025-10-17

**Authors:** Nicky Stanley, Christine Barter, Kelly Bracewell, Khatidja Chantler, Nicola Farrelly, Emma Howarth, Katie Martin, Helen Richardson Foster, Rhiannon Tudor Edwards, Eira Winrow

**Affiliations:** 1 School of Health, Social Work and Sport, 6723 University of Central Lancashire, Preston, UK; 2 5289 Manchester Metropolitan University, Manchester, UK; 3 School of Psychology, University of East London, London, UK; 4 School of Medical and Health Sciences, 1506 Bangor University, Bangor, UK

**Keywords:** domestic abuse, domestic violence, interventions, services, survivors

## Abstract

Concerns that community-based domestic abuse (DA) services are not always accessible or responsive prompted two new interventions across five sites in England. The evaluation used service data, outcome measures, staff interviews, and surveys, and 98 interviews with survivors and children. A Social Return on Investment analysis was completed. Survivors described services as empowering: support was flexible and personalized. Positive change in outcomes was found. However, 30%–40% of referrals were declined with confusion regarding risk levels and catchment areas. Increased provision of DA services could improve accessibility, but services need to build their community profile and strengthen links with health services.

## Introduction

Specialist domestic abuse (DA) services in the United Kingdom have grown substantially over the last 50 years. From their beginnings in the refuge movement, they have aimed to be responsive to their users, and many include DA survivors among their staff and management (Hague, 2021). The greater part of specialist DA services in England and Wales is now community rather than refuge-based (Domestic Abuse Commissioner, 2021), and the client group has grown to embrace children, male victims and perpetrators in addition to female survivors. However, there are concerns that services continue to be difficult to locate and access, especially for survivors with complex/multiple needs (House of Commons Home Affairs Committee, 2018). A recent survey of over 4,000 DA survivors in England and Wales (Domestic Abuse Commissioner, 2021) found that over half had found it quite/very difficult to access help. Some of this difficulty may reside in the secrecy that has traditionally surrounded DA. Specialist services, in common with DA itself, have been hidden from public view and, while this may have protected victims from abusive partners, it has contributed to low visibility and may have reinforced the stigma surrounding DA. However, while prevalence figures for England and Wales show no recent significant change, with 4.8% of adults experiencing DA in the year ending March 2024 (Office for National Statistics (ONS), 2024), demand for DA services was on an upward trend at the time of this study (ONS, 2022), which coincided with the global pandemic. Women (the predominant users of DA services) seeking support from DA services are known to exhibit high levels of mental health need (Ferrari et al., 2016) and specialist DA services in the United Kingdom report meeting increasingly complex/multiple needs as other frontline services have retreated and need has intensified under austerity measures and during the COVID-19 pandemic (Stanley et al., 2021a).

A number of reviews provide evidence on the impact of community-based DA services. Rivas et al.'s (2016) systematic review of DA advocacy services found no clear proof of the effectiveness of intensive advocacy services but suggested that brief advocacy could offer short-term mental health benefits and reduce abuse, especially in pregnancy and for less severe cases. Subsequently, Rivas et al.'s (2019) realist review reported low confidence in pregnancy or having children as a significant mediator of outcomes, but focused on core principles for advocacy services. These included the strength of the therapeutic alliance and the importance of the setting and community in which the intervention was delivered. Hackett et al.'s (2016) meta-analysis of research assessing mental health interventions for domestic violence survivors and their children found that the interventions studied had a significant effect on the well-being of participants. While the majority of relevant studies included in these reviews were undertaken in North America, some UK studies of community-based services have identified positive outcomes. Ferrari et al.'s (2018) controlled trial in the United Kingdom found that eight sessions of a psychological intervention delivered by trained DA advocates produced improved mental health outcomes for survivors in both community and refuge settings. Howarth and Robinson's (2016) multi-site evaluation of Independent Domestic Violence Advocates (IDVAs) found that intensity of delivery and number of community resources accessed were related to positive survivor outcomes. IDVAs provide short-term interventions for those assessed as high risk; the community-based services studied here included IDVA services but also worked with DA survivors for whom risks were not immediate or high and whose needs arose largely from the long-term impact of DA on them and/or their children.

This study reports on an independent evaluation of community-based interventions for DA survivors across five sites in England undertaken between 2017 and 2021. Funded by the Big Lottery's Women and Girls Initiative, two leading DA organizations, Women's Aid Federation England (WAFE) and SafeLives (SL), cooperated over 5 years to develop and deliver respective interventions for women and children experiencing DA. The evaluation aimed to identify the accessibility and responsiveness of community-based services delivered directly to DA survivors, whether they achieved change, and the barriers encountered.

In this paper, we use the terminology of domestic abuse (DA) as this is the language currently used in UK legislation and policy. The term community-based refers to non-residential services providing advocacy, advice, counseling, and groupwork. As is the case for most community-based DA services in the United Kingdom, interventions were delivered by third sector or voluntary organizations operating outside criminal justice, health, and child protection statutory services.

## The Interventions

The two organizations consulted extensively with survivors to develop and implement two different multi-component programs. WAFE's Change that Lasts intervention was delivered by established services affiliated to WAFE in three sites in North-East, the Midlands, and South-East England. In contrast, the SafeLives Co-Designed Pilots (SLCDPs) were newly commissioned from local providers in East and South-East England.

WAFE's program aimed to increase responsiveness to DA services at three levels. At the first two levels—community and frontline professionals—the program provided training to community ambassadors/volunteers and to social care and housing practitioners. At the third level of specialist DA services, the VOICES intervention delivered training and organizational support aimed at ensuring more responsive and trauma-focused services for survivors, and we report on this here. Evaluations of other Change that Lasts interventions are reported elsewhere (Stanley et al., 2021b).

The two SLCDPs offered a suite of interventions for survivors assessed as being at “medium risk,” their children and perpetrators, which together constituted a “whole family” approach (Stanley and Humphreys, 2017). IDVA services, a service for survivors with complex needs, and a recovery service were also provided. Survivors moved between interventions as needs changed or were reduced. This multi-component service was designed to respond to the diversity of needs while keeping survivors within one organization, so avoiding referral elsewhere.

## Methods

The study adopted a mixed methods approach that sought to identify change, its mechanisms and conditions, and the barriers identified. In line with Skivington et al.'s (2021) framework for complex evaluations, we included diverse stakeholder perspectives—survivors and their children, staff, volunteers, program managers and other professionals were interviewed—and the relevance of context (Pawson and Tilley, 1997) was acknowledged with consultations with local stakeholders and site profiles completed in all five sites (Stanley et al., 2021b).

Service data were cleaned and analyzed to provide information on referral pathways and service use. Impact for adult survivors was measured using both service data and a common outcome measure designed by the research team. This measure was administered at three or four time-points (according to the different lengths of the interventions delivered) and included a mixture of tested measures and bespoke questions to address the following outcomes:

- Well-being: Short Warwick-Edinburgh Mental Well-Being Scale (SWEMWBS) (Stewart-Brown et al., 2009).
- Safety: Evaluators’ own scale adapted from Well-being and Safety questions, part of the Space for Action scale in Kelly et al. (2014) scale.
- Coping and Confidence questions adapted from REVA (Responding Effectively to Violence and Abuse) (Kelly et al., 2014) scale.
- Health: EQ-5D-3L (EuroQol Research Foundation, 2018) and visual analogue scale (VAS thermometer).
- Perceptions of service.

Program staff facilitated recruitment, and 98 survivors and children were interviewed (or participated in focus groups if taking part in group programs) about their perceptions and experience of services. Some early interviews took place in person, but most were completed by telephone or online due to COVID-19 restrictions. All interviews were recorded, transcribed, coded in NVivo software, and analyzed thematically (Braun and Clarke, 2006).

A survey of program staff and interviews with eight senior managers were completed in the final year of the program, exploring their perspectives on their organizations and practice. Social Network Analysis (Gillieatt et al., 2015; Sabot et al., 2017) to identify networks and patterns of influence was undertaken with questionnaires completed by 27 staff across the five sites, followed up by two online surveys exploring network ties and referral patterns.

The evaluation included a Social Return on Investment (SROI) analysis (Nicholls et al., 2009) incorporating outcome and survey data, interviews, and findings from consultation groups with external stakeholders. WAFE and SafeLives provided data on costs to populate an SROI Impact Map: a spreadsheet exploring the relationship between the inputs (the resources used for the programs), the outputs (the programs themselves), and outcomes. National data from the HACT Social Value Bank (Trotter et al., 2014) was utilized to demonstrate where spending might increase or decrease.

### *Ethical Issues and Survivor Involvement*

The University of Central Lancashire gave ethical approval for the study. In line with research guidance (Women's Aid, 2020), the safety and well-being of participants were prioritized throughout. All participants were provided with appropriately formatted information about the study and gave informed consent for recorded interviews. Numerical identifiers were assigned to ensure anonymity, and interviews were planned to ensure that they could take place safely without being overheard. Interviewees were directed to sources of support if required. A Survivors’ Advisory Group advised on the design of research tools and interpretation of findings, and assisted recruitment of survivor researchers who received training, preparing them to contribute to data collection and analysis. Not all survivor researchers had sustained involvement throughout the evaluation, but those who did provided valuable insights on data analysis and interpretation.

### *Limitations*

The COVID-19 pandemic impacted program implementation, research recruitment, and practice. Delivery organizations remained operational throughout national lockdowns, but some planned aspects of the program were only partially implemented. Staff had less capacity to support recruitment and completion of outcome measures. Most interviews had to be undertaken online or by telephone. However, there were opportunities to study the shift to remote delivery of services, and findings are reported elsewhere (Richardson-Foster et al., 2022).

The researchers drew on service records to provide demographic information and details of need at service entry. The two interventions used different recording and monitoring systems; there were also gaps and omissions in records and difficulties in matching service data with the outcome measures used for the evaluation. While 2 years’ worth of service data was collected for those using SafeLives services, implementation delays meant that only 12 months’ worth of service data could be captured for WAFE service users.

## Findings

### *Implementation*

Training was key to both interventions, with WAFE staff receiving training and tools to assist with implementation of the VOICES approach while SLCDP staff and managers received extensive training in the use of various tools and approaches at start-up. The second phase of WAFE's VOICES involved work with local managers and management boards on organizational culture, leadership, and supervision. Both organizations also provided training for other local professionals as a means of raising awareness of DA and ensuring relevant referrals for their services.

There were considerable implementation challenges. VOICES was a new approach that was not introduced until Year 3 of WAFE's Change that Lasts program, and implementation was affected by COVID-19 restrictions. As noted above, this limited the evaluation's data collection, which was undertaken predominantly in two sites, as only baseline data with no follow-up data were available for one site.

The SLCDP services started up in 2018, and we therefore report on data collected over 24 months. Staff recruitment and retention were a particular challenge for these services, which experienced high staff turnover: 17 staff resigned or left their posts from October 2018 to November 2020. Nine posts were vacant for more than 1 month in the same period. Reasons for staff turnover included staff being offered longer-term contracts and higher salaries elsewhere (SLCDP salaries failed to match those offered elsewhere in one region) and, for a small number, feeling “burnt out” due to high caseloads.

The involvement of “experts by experience” in the design and development of interventions aimed to ensure that services were congruent with users’ needs. However, the planning required to ensure the meaningful participation of these groups required resources and extended the design period. The national remit of these groups also meant that local conditions and context (such as relevant wage levels) were not always taken into account.

### *Survivors’ Profiles*

Profiles for those using the two services are reported separately as the service records utilized covered different time periods. The vast majority of those using both services were women.

#### WAFE Service Users

Over half (62%) of those using WAFE services were not living with the perpetrator at the time of referral. Table 1 shows that 60% were aged 26–45. Almost three-quarters (73.4%, *n* = 972) had children, with 2,821 children recorded in service records.

**Table 1.** Demographic Details of WAFE Survivors (*n* = 2,125) in Three Services, November 2019-November 2020.

	*N*	%
**Gender**		
Female	2045	96.2
Male	65	3.1
Intersex	1	0.0
Do not know	14	0.7
**Age (years)**		
0–15	2	0.1
16–25	378	17.8
26–35	765	36.0
36–45	507	23.9
46–55	256	12.0
56–65	77	3.6
66–75	38	1.8
76+	16	0.8
Missing data	86	4.0
**Ethnicity**		
White	1513	71.2
Mixed/multiple ethnic groups	12	0.6
Asian/Asian British	52	2.4
Black/African/Caribbean/Black British	20	0.9
Another ethnic group	13	0.6
Do not know/declined/not asked	515	24.2
**Parenting status^a^**		
Child(ren) Yes	972	73.4

a This information was not available from one of the three WAFE services included in the study, so these figures are based on information provided by two services only.

74% were not living with the perpetrator when referred, although nearly a quarter (24%) lived with the perpetrator full-time or intermittently. Table 2 shows that nearly 74% of survivors were aged 26–45 years old. Children were involved in 84% of cases, although not all these children engaged with the SLCDP services.

**Table 2.** Demographic Details of SLCDP Survivors and Children and Young People in Two Services, November 2018–December 2020.

	SLCDP Survivors		SLCDP Children and Young People	
	*N*	%	*N*	%
**Age (years)**				
0–4			11	4.1
5–7			72	26.7
8–11			111	41.1
12–17			76	28.1
17–25	53	11		
26–35	199	41.4		
36–45	158	32.8		
46–55	52	10.8		
Over 55	19	4		
**Ethnicity**				
White	400	83.1	225	83.3
Mixed/multiple ethnic groups	12	2.5	31	11.5
Asian/Asian British	27	5.6	5	1.9
Black/African/Caribbean/Black British	17	3.5	6	2.2
Other ethnic group	6	1.3	2	0.7
Not disclosed/don't know	19	4.0		
**Are children involved in the case?**				
No	78	16.2		
One or more	403	83.8		
**Total**	**481**	**100**	**270**	**100**

Tables 1 and 2 show that most survivors from both services were of White British heritage, although this data was missing from service records for almost a quarter of WAFE survivors. While ethnicity figures were in line with those for the local population in most sites, the WAFE site in a Midlands city recorded only 4.5% Black and Minoritized women using its services compared to the local authority's Black and Minoritized population of 36% reported in the 2021 Census (ONS, 2022). While Black and Minoritized survivors may have used specific local DA services targeting their communities (known as “By and For Services”) in preference to the VOICES service, this disparity strongly suggests that accessibility of these services to Black and Minoritized communities could be increased.

WAFE survivors had experienced multiple forms of DA; the most commonly recorded was emotional abuse (99%, *n* = 2103), followed by physical abuse (61.6%, *n* = 1310), jealous/controlling behavior (57.1%, *n* = 1214), and surveillance/harassment/stalking behaviors (39.5%, *n* = 840). Survivors had experienced abuse for an average of 7 years prior to accessing the service.

#### SLCDP Service Users

Data for the two SLCDP services covers 2 years, but these newly established services worked with survivors for longer periods than the WAFE services, so the numbers are considerably lower.

The majority of SLCDP survivors had experienced DA in the past 12 months, and nearly one third (29%) had experienced multiple forms of DA (physical violence, sexual violence, stalking, and coercive control), with most describing the severity level as either standard or moderate. Controlling, coercive, and jealous behaviors were experienced by two-thirds (67%) of all survivors in the sample. A third of survivors had experienced DA for 1–4 years, and a further 41% for over 5 years.

The SLCDP services targeted children and young people aged 4–17, and just under half (41%) of the 270 receiving services were aged 8–11 years (*M*_age_ = 9.48, *SD* = 3.21). In common with their mothers, most were of White ethnicity (83%). Service records identified 42% as currently exposed to DA at home.

Although SLCDPs aimed to work with perpetrators, the number of predominantly White male perpetrators using the service over the 2 years of the evaluation was low (11.4% of cases included work with perpetrators). The evaluation collected limited data on perpetrators since the study focused on interventions for women and children.

### *Pathways Into and Through Services*

Service records provided a picture of survivors’ pathways to services. The two programs drew on contrasting referral sources. WAFE services received the majority of their referrals from the police or MARACs (Multi-Agency Risk Assessment Conferences). WAFE practitioners reported using some of the tools introduced in their VOICES training to structure conversations with external agencies and improve the quality of referrals. Nevertheless, service records for the 12-month evaluation period showed that 40.4% of referrals to community-based services were rejected. Waiting lists could be lengthy: in one site, almost a quarter of those accepted had to be placed on the waiting list (22.9%, *n* = 64).

Service use was generally short-term, on average lasting between 1.73 and 3.27 months, and this goes some way to explaining the considerably higher number of cases the three WAFE organizations worked with compared to SLCDP services. Roughly half of all case closures were planned. The most common reason for unplanned closure (38% of all cases closed over 12 months) was that the survivor had never engaged or had disengaged.

The bulk of SLCDP referrals came from either children's social care or from other DA or sexual violence services that had assessed the survivor as ineligible for their service or no longer appropriate due to a change in their circumstances. In some cases, this eligibility concerned the level of risk assigned to a case since all independent community-based DA services across the two sites were categorized as addressing medium-risk cases.

The two SLCDPs recorded 399 (30.5%) declined referrals from a total of 1,307 received across the 2 years of the evaluation period. This is substantially lower than the 45% reported in WAFE's 2020–2021 annual survey of DA services across England (Women's Aid, 2022). In one site, the main reason for declined referrals (49%) for one of the two services was that they came from outside the service's catchment area, which was relatively small; this was a rarer reason for declining referrals in the other service. High-risk referrals accounted for 21% of declined referrals in one service and 28% in the other.

The SLCDP services were restricted to survivors classified as “medium risk,” and risk levels could shift while a survivor was waiting for or receiving the service, resulting in “shutting and opening the service door” (Senior Manager 3, SLCDP). Another manager highlighted the discontinuity that occurred when risk levels changed and survivors were referred on to another service addressing a high level of risk: “…for victims, that then go from medium risk and having lots of support and engaging well and building a rapport with our team, to then have to move to a different team … that does have an impact” (Senior Manager 5, SLCDP).

Staff shortages in one SLCDP site led to waiting lists, which were described by staff as reducing confidence in the service. Some survivors reported a lengthy wait, which the service acknowledged and aimed to manage:

It was about six months but [workers] did call me every couple of weeks, just for a check and see how things were, and whether I needed anything whilst waiting. So, I wasn’t forgotten. (Survivor 3.11, SLCDP)

Adult survivors’ average length of use of SLCDP services was 7.5 months, and this relatively lengthy engagement may also have contributed to waiting lists in one site. However, some survivors interviewed would have preferred a longer engagement with the service. The majority of children (60%; 112 of 187) received a service for over 6 months. Over a quarter of survivors’ exits were unplanned (96 of 362 closed cases), with disengagement from the service being recorded as the main reason. Exit data for the 187 children whose cases were closed in this period showed that 18% had an unplanned exit.

### *Achieving Responsive Services*

The majority of survivors interviewed from both interventions were highly positive about the services received and described how engagement was achieved through staff's approachability and by providing sufficient time and opportunities for women to talk:

Whenever I telephoned, there was always someone on the other end of the phone… And they talk to you for as long as you want to be on the phone for. (Survivor 3.10, WAFE)

One of the key principles informing both programs was the need to trust and strengthen women's own understanding of what they needed and when: not “doing to,” but collaboratively “doing with” and “alongside.” Survivors recognized and valued this approach:

…they didn’t actually tell us what to do, if you know what I mean. They like kind of advised us and that's kind of like what I prefer. I don’t like to be told what to do. (Survivor 2.7, WAFE)

Survivors often required support with court cases or contact issues and appreciated input that was delivered in a timely way in response to these needs:

The first psychological assessment, which the court ordered, I suddenly got really upset and I got really down… And I rung [my worker] and she talked me through it and she gave me some advice … I definitely needed her then. (Survivor 1.5, SLCDP)

Flexibility regarding the location of face-to-face meetings also assisted engagement, and mothers noted the value of this for children as well as for themselves:

that was a big thing that [CYP Worker] was able to meet elsewhere … which helped the children no end, especially [my son] because he was away from the environment that he didn’t like … so he could open up more… (Survivor 1.9, SLCDP)

The multi-component SLCDPs facilitated support packages tailored to individual and family needs. This could include individual and group work, work targeting parenting, DA recovery work, and work with children. This personalized approach was valued:

…they were all at the right time for where I’m at and this has helped me like at this stage of where I’m going, it's helped me sort of put into practice things I’ve learned. (Focus Group 1.1, SLCDP)

Women appreciated regular and consistent contact with workers who they felt cared about them: “I really feel like she cares” (Survivor 1.15, SLCDP). Authenticity was important, and this was enhanced when workers had relevant experience:

…some of them that work there, have actually been through what we’re all going through. It's like, they understand a hell of a lot more. (Survivor 2.7, WAFE)

Women also noted when staff worked well together as a team. The multi-component service offered by SLCDP was perceived as well-coordinated, with one survivor describing staff as “all singing off the same sheet” (Survivor 2.5, SLCDP). The offer of a flexible, personalized service for children was sometimes an incentive for mothers to engage with the service:

She explained everything that they could help, for me, and for the children. And always individually… It was like, we can offer this for [older child], we can offer this for [younger child], and I thought, it was always really personalised. (Survivor 2.7, SLCDP)

### *Barriers to Delivering Responsive Services*

Most of the barriers to delivering responsive services identified by survivors concerned implementation factors such as staff turnover, waiting lists, and age restrictions for children accessing the SLCDP service. These factors also impacted staff's experience: half the staff surveyed across both programs described workloads as often/always too heavy, and staff shortages will have contributed to this.

Some survivors would have liked more information about services earlier, and this was particularly evident in respect of the SLCDPs, which offered a wide menu of interventions.

The ambition and complexity of the SLCDPs meant that some services were not delivered as intended. Staff recruitment and retention problems resulted in both the perpetrator's service and that designed for survivors with complex needs working with small numbers. Waiting lists for one group had knock-on effects for other groups and impacted the ambition to deliver an integrated whole family service:

…we can’t start with them (perpetrators) until we start with the victim. So that means that we will lose some perpetrators but by the time the victim and the children can both get a service, that perpetrator might well have lost motivation. (Staff 1, SLCDP)

### *Meeting Complex/Multiple Needs*

The complexity of survivors’ needs was evident across both organizations. WAFE service data showed 63% of survivors had multiple needs such as mental health, physical health, alcohol and/or drug issues; mental health need was most frequently recorded (55.6%; *n* = 755). At baseline, SLCDP service data showed 12% of survivors had mental health needs, 10% had physical, neurological, and/or progressive illnesses, 8% had alcohol problems, and 5% drug misuse. However, the outcome measures completed at baseline with 188 SLCDP survivors showed a more challenging picture with low well-being scores on the SWEMWBS for most survivors: the mean score was 21.10 with a range of 11.25–35. Similarly, on the EQ-5D-3L measure, SLCDP survivors at baseline experienced worse health states than the general population.

Women with complex/multiple needs constituted the groups that both WAFE and SLCDP staff found more difficult to engage with: WAFE staff flagged up challenges with women with substance misuse problems, while SLCDP staff considered women who were homeless or those who had mental health problems most difficult to engage.

These findings suggest the value of close links with other specialist services, especially mental health and substance misuse services. However, the Social Network Analysis, which examined the extent and strength of the organizations’ ties with other local services, found weak and infrequent links with health services for all four services whose staff participated in this element of the study. While one of the SLCDP services had strong ties with children's social care, both in terms of receiving referrals and providing advice, and one of the WAFE services had frequent interactions with social care and justice/legal services, none had regular or strong contact with health services.

### *Adult Survivor Outcomes*

The direction of change was positive on most of the outcomes measured, with improvements found on measures of safety, coping, and confidence and mental well-being.

In the WAFE sites, the pandemic impacted the completion of outcome measures over three time-points, with the result that we are only able to report on short-term change at 6–8 weeks (T2) from baseline (T1). Although, as noted earlier, these were short-term services by comparison with the SLCDPs, given the small sample with high levels of attrition, these findings are indicative only. While no significant change was found in safety, positive responses increased between T1 and T2 for 10 of the 11 questions on coping and confidence. The 91 survivors who responded to the seven mental well-being questions at T1 had a mean average sum score of 22.72, which is slightly above the mean of 21.10 for all survivors found in WAFE service records, but again is just below the UK population norm for women (23.6) (Ng Fat et al., 2017). At T2, 50 survivors completed the seven questions, producing a slightly higher mean average sum score of 24.41.

SLCDP outcomes were captured over 2 years, and 57 survivors completed outcome measures at both baseline and 6 months follow-up (T3). Safety improved at 6 months from baseline, although changes were only statistically significant with respect to safety at home and neighborhood. Between baseline and service exit, there were moderate or small statistically significant improvements for all six safety questions. Measures of coping and confidence showed improvements at 6 months on nearly all items, with the change reaching statistical significance on six dimensions. At service exit, four of these dimensions showed statistically significant improvements, all with small effect sizes.

**Figure 1.**
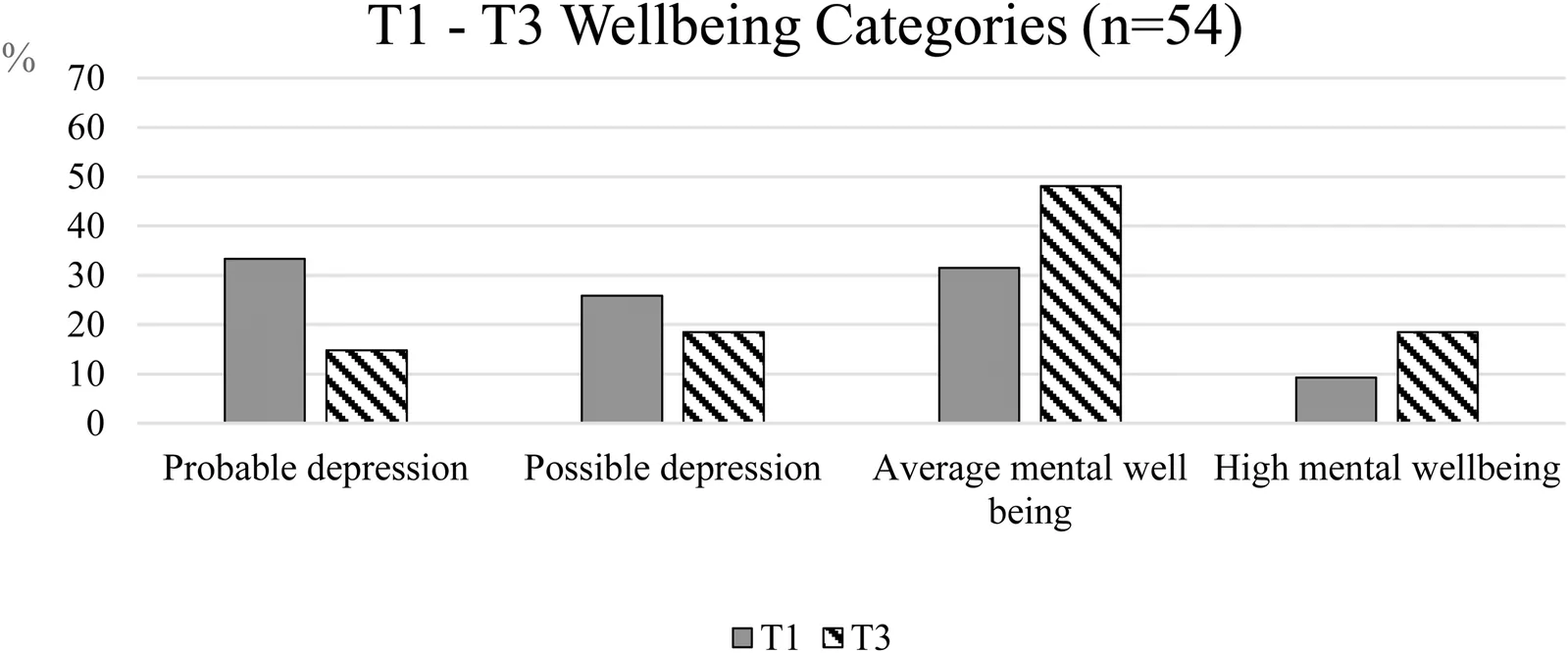
Mental Well-being in SLCDP Survivors at T1 (Baseline) and T3 (6 Months).

Figure 1 shows the significant levels of change found in respect of SLCDP survivors’ mental well-being 6 months from baseline: women moved out of the probable and possible depression categories into average and high mental well-being. The mean baseline score of 20.71 rose to 23.3, a statistically significant increase of 2.54, *t*(53) = −4.254, *p* ≤ .001). This shift produced a mean still lower than the national average for women of 23.6 (Ng Fat et al., 2017), reflecting the particularly low levels of mental health for these survivors at baseline.

Using an approach from Contribution Analysis (Mayne, 2011), survivors were asked at T2 and T3 whether they attributed any improvements across the four domains of safety, coping and confidence, well-being, and health to the program. At T2, most WAFE survivors who had experienced improvements were very positive about services, with between 60% and 73% attributing their improvements either mostly or entirely to the VOICES service. At both T2 and T3, SLCDP survivors reported improvements and either mostly or entirely attributed this change to the SLCDP service they had used.

### *Social Return on Investment*

**Figure 2.**
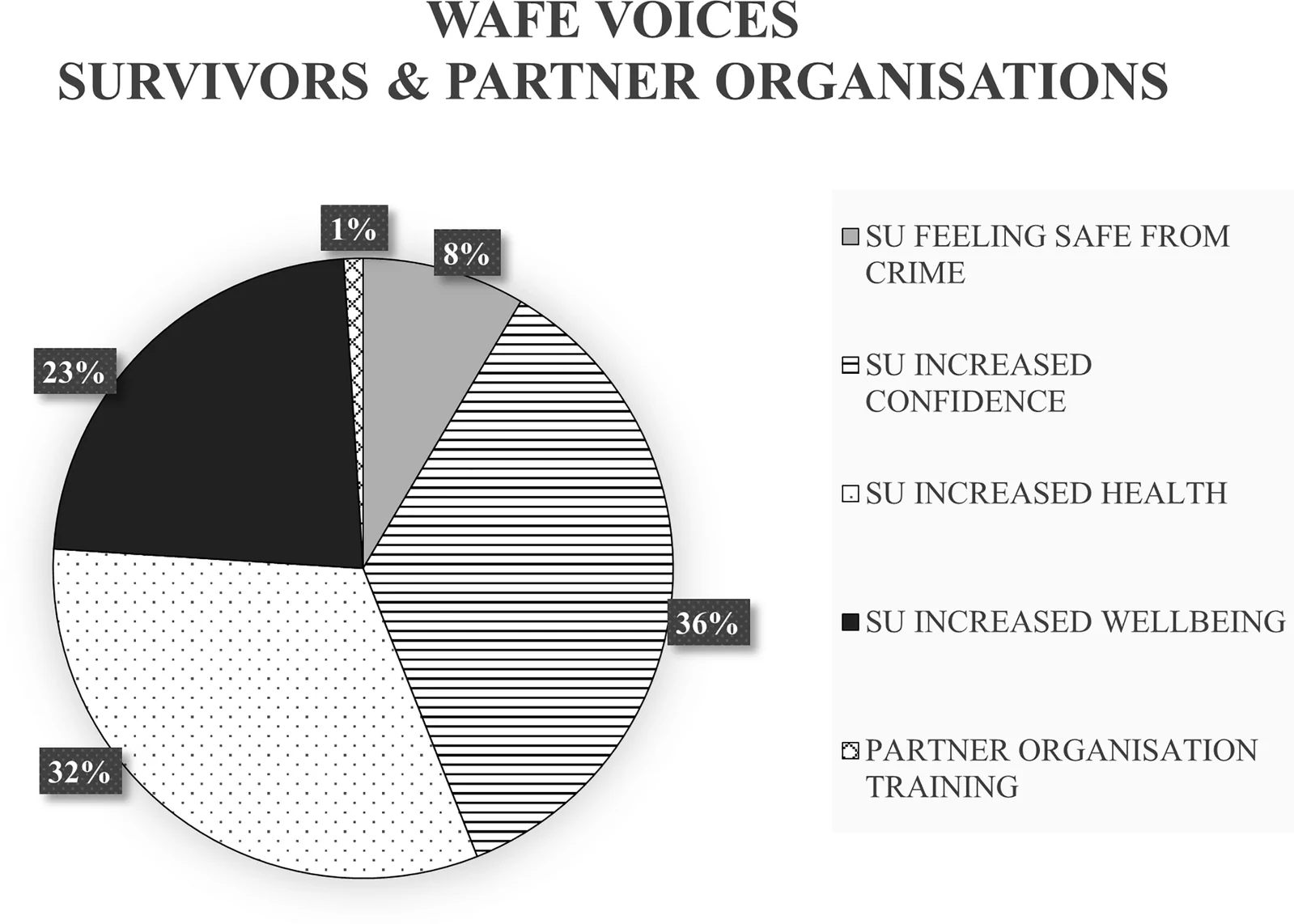
Elements of Social Value: WAFE VOICES.

SROI is reported in terms of the social value return on the financial investment, so for every pound invested, a social value figure can be reported. WAFE's VOICES intervention was calculated to generate a range of SROI ratios with a value between £4.51 and £7.37, with a base-case scenario or mid-range figure of £5.50 for every pound invested. Figure 2 shows the breakdown of elements used to produce this figure. Survivors (SU) were the group that benefited most from the WAFE intervention, particularly in the areas of improved health, well-being, and confidence.

The average length of time survivors had experienced DA prior to accessing VOICES was 7 years. Using the Home Office annual unit cost of DA in the United Kingdom of £34,015 per person (Oliver et al., 2019), the potential cost of DA in the 109 cases where outcome measures were completed would be £3.7 m, but many of these survivors experienced DA over 7 years, so this can be considered a conservative estimate. The data collected for the WAFE intervention demonstrated potential to contribute not only to the safety of survivors but also to cross-sector costs.

**Figure 3.**
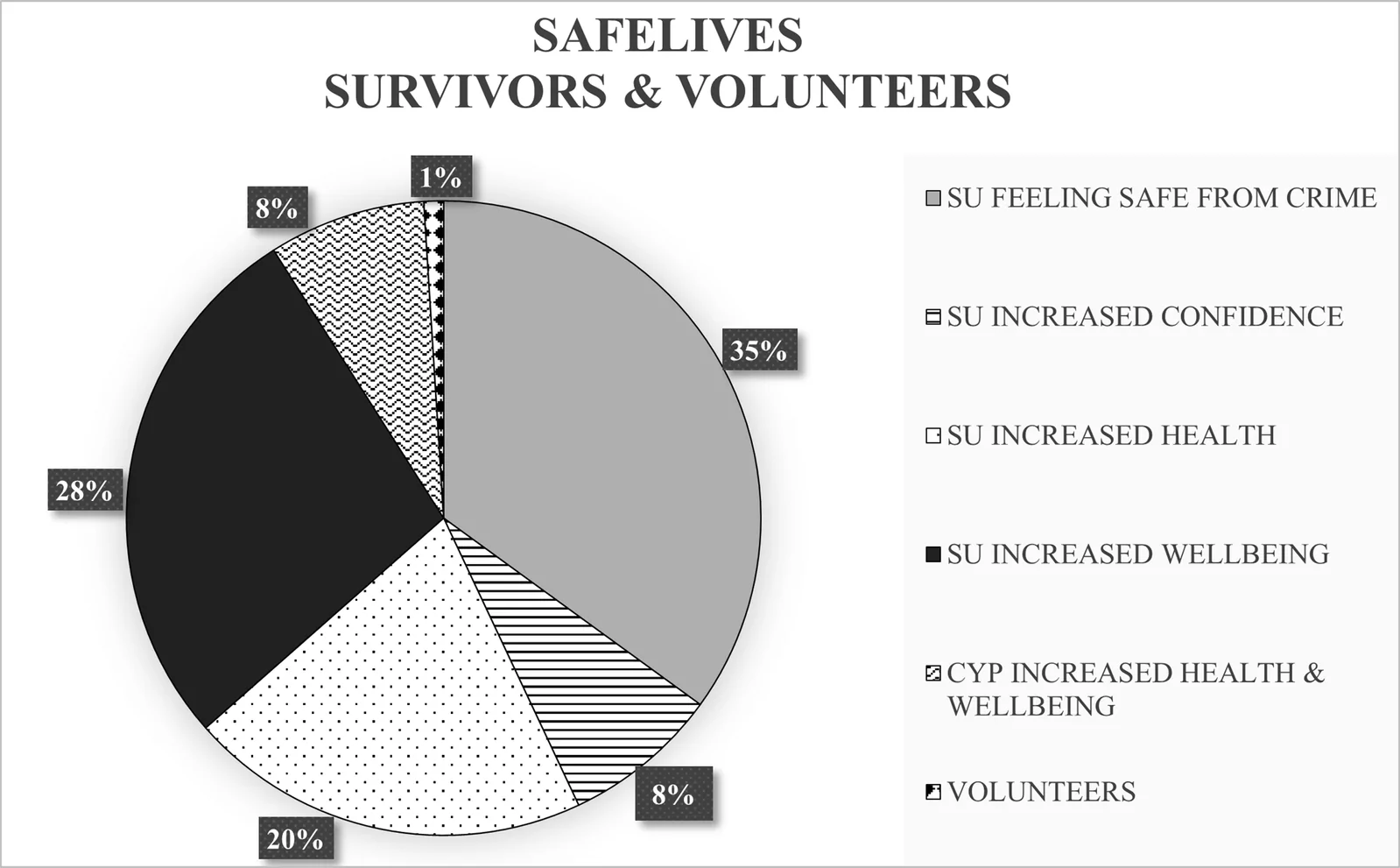
Elements of Social Value: SafeLives Co-Designed Pilots.

Figure 3 shows the breakdown of the elements generating the SLCDP SROI figure. Survivors (SU) benefited from the greatest generated social value with increased improvements in health, well-being and feeling safe from crime. Their children (CYP) benefited in terms of improved health and well-being, and a small proportion of the social value benefited volunteers contributing to the design and delivery of the service. The SLCDPs generated a range of social return on investment between £4.18 and £6.75, with a mid-range figure of £5.36 for every pound invested.

## Discussion

This study examined whether and how two different approaches to delivering DA services succeeded in delivering accessible and responsive services, which also achieved impact. While the evidence for the responsiveness and impact of the services studied was encouraging, challenges were identified in respect of achieving accessibility.

The broadening remit of community-based DA services needs to be acknowledged here. While DA refuges are primarily focused on providing safety and immediate support, community-based services are working with both the immediate and long-term health and social consequences of DA, as well as ensuring safety. Although these services continue to receive referrals from the police and accept high-risk cases from MARACs, many, as demonstrated by the SLCDPs, are now taking on large numbers of referrals from children's social care, are working directly with children, and on parenting issues. The high proportions of women with children using both services in this study confirm the finding of Ravi et al.'s (2022) systematic review of facilitators of help-seeking for adult IPV survivors in the United States that children's needs with respect to DA can act as a motivation for seeking support. In our study, SLCDP mothers described the offer of a service for their children as an incentive for engagement.

Despite a context of high demand for DA support, both services were recording high rates of declined referrals. While these were in line with or lower than national figures for 2020–2021 (Women's Aid, 2022), it is of concern that so many referrals were declined. Newly established services, such as the SLCDPs, which need to forge referral pathways from scratch, may require time to attract appropriate referrals. Nevertheless, the more established WAFE services also recorded high rates of declined referrals. While in 2020–2021, the impact of the pandemic on services will have restricted capacity, Women's Aid national estimated figures show a steady trend of increasing declined referrals predating the pandemic with 24.3% of referrals to community-based services declined in 2016/17 (Women's Aid, 2018) rising to 41.1% in 2019/20 (Women's Aid, 2021). Shortfalls in provision are likely to play a key part here, but there may also be a mismatch of expectations and service offer. Both survivors and those referring them to DA services need transparency about what is on offer for whom: labeling services according to risk levels means little to those using them and may lead to frustrations and fluctuations in service delivery when assessments of risk change or vary, as they frequently do (Almond et al., 2017). Ravi et al.'s (2022) review identified awareness of DA services and knowledge of service processes as a requisite for approaching services. Service accessibility for Black and Minoritized women also requires more consideration.

While adopting contrasting models, both organizations succeeded in offering responsive interventions, and this achievement may reflect survivors’ involvement in service design and planning. Women reported being able to exercise choice and control in their interactions with these services, and, in this respect, these DA services promoted empowerment and enabled those who had experienced coercion in their intimate relationships to experience a sense of control. In common with other studies (Kulkarni et al., 2012; Rivas et al., 2019; Paphitis et al., 2022), this study found that DA survivors valued the relational aspects of service delivery and appreciated services that could be tailored to their individual needs.

We have not attempted to systematically compare outcomes for the two services studied here since the amount and quality of outcome data captured varied. While both services showed positive change on outcome measures, significant improvement in respect of mental well-being was found for the SLCDPs, and this may reflect the longer period of service use (see also Howarth and Robinson, 2016). Notably, those survivors participating in the Domestic Abuse Commissioner's (2021) survey wanted support over longer periods of time. Service duration merits further study, especially given the cost implications of longer interventions.

The findings on mental health outcomes are consistent with Ogbe et al.'s (2020) review, which identified good to moderate evidence for survivor-focused advocacy's positive impact on mental health. Paphitis et al.'s (2022) realist review also noted that flexible interventions tailored to individual need, like those evaluated by this study, may produce more meaningful changes in outcomes. Their review also flagged the value of multi-layered interventions that addressed complex/multiple needs and forged relationships with other service providers (Paphitis et al., 2022).

Both organizations were working with survivors with complex/multiple needs, especially in respect of mental health and substance misuse. Given this, it was concerning that the Social Network Analysis found limited contact between these specialist DA services and health services. Health professionals are often the first recipients of DA disclosures (Domestic Abuse Commissioner, 2021) as well as a source of expertise in meeting health needs. There have been some UK interventions aimed at improving referral pathways and strengthening health services’ response to DA, including the IRIS primary care intervention (Feder et al., 2011) and hospital-based IDVAs (Halliwell et al., 2019). However, initiatives linking community-based DA services to mental health and substance abuse services remain sparse in the United Kingdom. The limited engagement of health services in DA, together with competing priorities, might explain why the links between the specialist DA sector and health remain underdeveloped.

Both programs generated levels of social value consistent with that identified by other economic evaluations of interventions delivered by specialist community-based DA services (Solace, 2015; Selsick and Atkinson, 2016). In contrast, Dowrick et al.'s (2022) SROI analysis of the UK's IRIS program reported a considerably higher SROI of £10.71, but IRIS is a training and referral pathway in primary care that draws heavily on established health service resources.

## Conclusion

This study found that ambitions to deliver accessible and responsive DA services were partially met. The services evaluated were responsive to survivors’ needs and produced social value and positive outcomes, significantly in respect of mental health. However, ambitions may be circumscribed by short-term funding, which, even for the 5-year span of these programs, restricted the time available for planning, development, and implementation and contributed to staff churn. The pandemic also contributed to these restraints.

Survivors’ routes to DA services addressing both immediate and long-term needs are becoming more diverse, and, if pathways to support are to be short and direct, DA services need to increase their community profile and promote their services in a way that is comprehensible to those who use and refer to them. Subsequent to the “Me Too” movement and the pandemic, DA has emerged as an issue attracting increasing public sympathy, and the sector's reticence about advertising its services widely may no longer be appropriate. However, increased funding needs to be available on a longer-term and less piecemeal basis for the sector to be able to move to this position. The Domestic Abuse Act 2021 placed a duty on commissioners in England and Wales to fund accommodation for DA survivors, but funding for community-based services remains fragile, and there is a risk that it may be diverted to meet the new duty in respect of accommodation.

This study also suggests that the aims of the UK's national guidance for health and social care (NICE, 2014), which exhorted health services to engage closely with local DA services, have yet to be fully realized. Given the high levels of health need that DA services are confronting, new strategies are required at the national and local levels to promote collaboration.
